# Safety and Efficacy of PD‐1/PD‐L1 inhibitors combined with radiotherapy in patients with non‐small‐cell lung cancer: a systematic review and meta‐analysis

**DOI:** 10.1002/cam4.3718

**Published:** 2021-01-19

**Authors:** Yichao Geng, Qiuning Zhang, Shuangwu Feng, Chengcheng Li, Lina Wang, Xueshan Zhao, Zhen Yang, Zheng Li, Hongtao Luo, Ruifeng Liu, Bing Lu, Xiaohu Wang

**Affiliations:** ^1^ The First School of Clinical Medicine Lanzhou University Lanzhou China; ^2^ Institute of Modern Physics Chinese Academy of Sciences Lanzhou China; ^3^ Lanzhou Heavy Ion Hospital Lanzhou China; ^4^ Department of Oncology The Affiliated Hospital of Guizhou Medical University Guiyang China; ^5^ Department of Oncology Guizhou Cancer Hospital Guiyang China; ^6^ Basic Medical College Lanzhou University Lanzhou China

**Keywords:** combined radio‐immunotherapy, meta‐analysis, non‐small‐cell lung cancer, programmed cell death protein‐1/programmed cell death ligand‐1 inhibitors, radiotherapy, systematic review

## Abstract

**Background:**

A combination of programmed cell death protein‐1 (PD‐1)/programmed cell death ligand‐1 (PD‐L1) inhibitors and radiotherapy (RT) is increasingly being used to treat non‐small‐cell lung cancer (NSCLC). However, the safety and efficacy of this approach remains controversial. We performed a systematic review and meta‐analysis to summarize the related research.

**Methods:**

We searched the China Biology Medicine, EMBASE, Cochrane Library, and PubMed databases for all the relevant studies. The Stata software, version 12.0 was used for the meta‐analysis.

**Results:**

The study included 20 clinical trials that enrolled 2027 patients with NSCLC. Compared with non‐combination therapy, combination therapy using PD‐1/PD‐L1 inhibitors and RT was associated with prolonged overall survival (OS) (1‐year OS: odds ratio [OR] 1.77, 95% confidence interval [CI] 1.35–2.33, *p* = 0.000; 2‐year OS: OR 1.77, 95% CI 1.35–2.33, *p* = 0.000) and progression‐free survival (PFS) (0.5‐year PFS: OR 1.83, 95% CI 1.13–2.98, *p* = 0.014; 1‐year PFS: OR 2.09, 95% CI 1.29–3.38, *p* = 0.003; 2‐year PFS: OR 2.47, 95% CI 1.13–5.37, *p* = 0.023). Combination therapy also improved the objective response rate (OR 2.76, 95% CI 1.06–7.19, *p* = 0.038) and disease control rate (OR 1.80, 95% CI 1.21–2.68, *p* = 0.004). This meta‐analysis showed that compared with non‐combination therapy, combination therapy using PD‐1/PD‐L1 inhibitors and RT did not increase the serious adverse event rates (≥grade 3); however, this approach increased the rate of grade 1–2 immune‐related or radiation pneumonitis. Subgroup analyses revealed that the sequence of PD‐1/PD‐L1 inhibitors followed RT outperformed in which concurrent PD‐1/PD‐L1 inhibitor and RT followed PD‐1/PD‐L1 inhibitor. Combination of stereotactic body RT or stereotactic radiosurgery with PD‐1/PD‐L1 inhibitors may be more effective than a combination of conventional RT with PD‐1/PD‐L1 inhibitors in patients with advanced NSCLC.

**Conclusion:**

Combination therapy using PD‐1/PD‐L1 inhibitors and RT may improve OS, PFS, and tumor response rates without an increase in serious adverse events in patients with advanced NSCLC. However, combination therapy was shown to increase the incidence of mild pneumonitis.

## Graphical abstract

Radiotherapy combined with PD‐1/PD‐L1 inhibitors in the treatment of advanced NSCLC showed clinical long‐term survival benefit and short‐term efficacy benefit, and might not increase the serious adverse events.

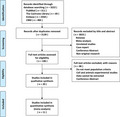

## 1 INTRODUCTION

Non‐small‐cell lung cancer (NSCLC) is a common malignant tumor associated with significantly high morbidity and mortality rates globally. Latest statistical data show that there will be 228,820 new cases of lung cancer and 135,720 deaths in 2020.^1^ Conventional treatments for NSCLC include surgery, chemotherapy, and radiotherapy (RT). Despite the progress in treatment of locally advanced and metastatic NSCLC using the aforementioned combination treatments over the past few decades,^2^ survival rates and local control rates of this malignancy remain unsatisfactory. Currently, immune checkpoint inhibitors (ICIs) have shown promising results as novel anti‐cancer drugs. ICIs are monoclonal antibodies that can eliminate T‐cell inhibitory pathways.^3^ Programmed cell death protein‐1/programmed cell death ligand‐1 **(**PD‐1/PD‐L1) inhibitors represent the most important and frequently used class of ICIs. Clinical trials are increasingly being performed in recent times to investigate the role of combination therapy using PD‐1/PD‐L1 inhibitors and RT for NSCLC.^4^ This combination is referred to as “combined radio‐immunotherapy.” However, conflicting conclusions have been drawn regarding the efficacy and toxicity profile of combined radio‐immunotherapy in patients with NSCLC. Some clinical studies support the administration of combined radio‐immunotherapy; for example, the PACIFIC study (a phase III, double‐blind multicenter clinical study with a 2:1 randomized control design)^5^ included patients with locally advanced NSCLC treated with durvalumab (a PD‐L1 inhibitor) followed by radiochemotherapy and reported significantly better overall survival (OS) and progression‐free survival (PFS) in this patient population than in those treated with radiochemotherapy alone. Adverse events (AEs) were similar between the groups. The KEYNOTE‐001 trial (a single‐center retrospective study) reported the same trend. A history of RT administered to any site can prolong PFS and OS in patients with metastatic NSCLC treated with pembrolizumab (a PD‐1 inhibitor) with acceptable toxicity.^6^ Two retrospective studies have shown that combination treatment with RT and nivolumab (a PD‐1 inhibitor) in patients with advanced NSCLC may improve OS and PFS without an increase in acute toxicity rates.^7^, ^8^ However, a few studies have reported contrasting results. For example, the PEMBRO‐RT trial showed that stereotactic body radiotherapy (SBRT) preceding pembrolizumab treatment for metastatic lesions did not improve long‐term survival compared with pembrolizumab alone in patients with metastatic NSCLC.^9^ Cho et al.^10^ reported that RT‐induced lymphopenia in patients with advanced NSCLC can reduce the efficacy of ICIs. A study performed by Bang et al.^11^ described higher overall toxicity following RT administration within 14 days of ICI treatment. A review by Li et al.^12^ suggests a high incidence of immune‐related or radiation pneumonitis in patients with advanced NSCLC who receive combination radio‐immunotherapy. As mentioned earlier, the efficacy and safety of combination therapy using PD‐1/PD‐L1 inhibitors and RT in patients with NSCLC remains controversial. The results of high‐quality meta‐analyses are increasingly being considered high‐quality evidence.^13^, ^14^, ^15^ Therefore, we performed a systematic review and meta‐analysis of the safety and efficacy of combination therapy using RT and PD‐1/PD‐L1 inhibitors for the treatment of patients with NSCLC.

## 2 MATERIALS & METHODS

This study was performed in accordance with the Preferred Reporting Items for Systematic Reviews and Meta‐Analyses guidelines.^16^, ^17^, ^18^ The protocol is available online at PROSPERO. A MeaSurement Tool to Assess systematic Reviews was used to assess methodological quality.^19^, ^20^

### 2.1 Search strategy

**FIGURE 1:**
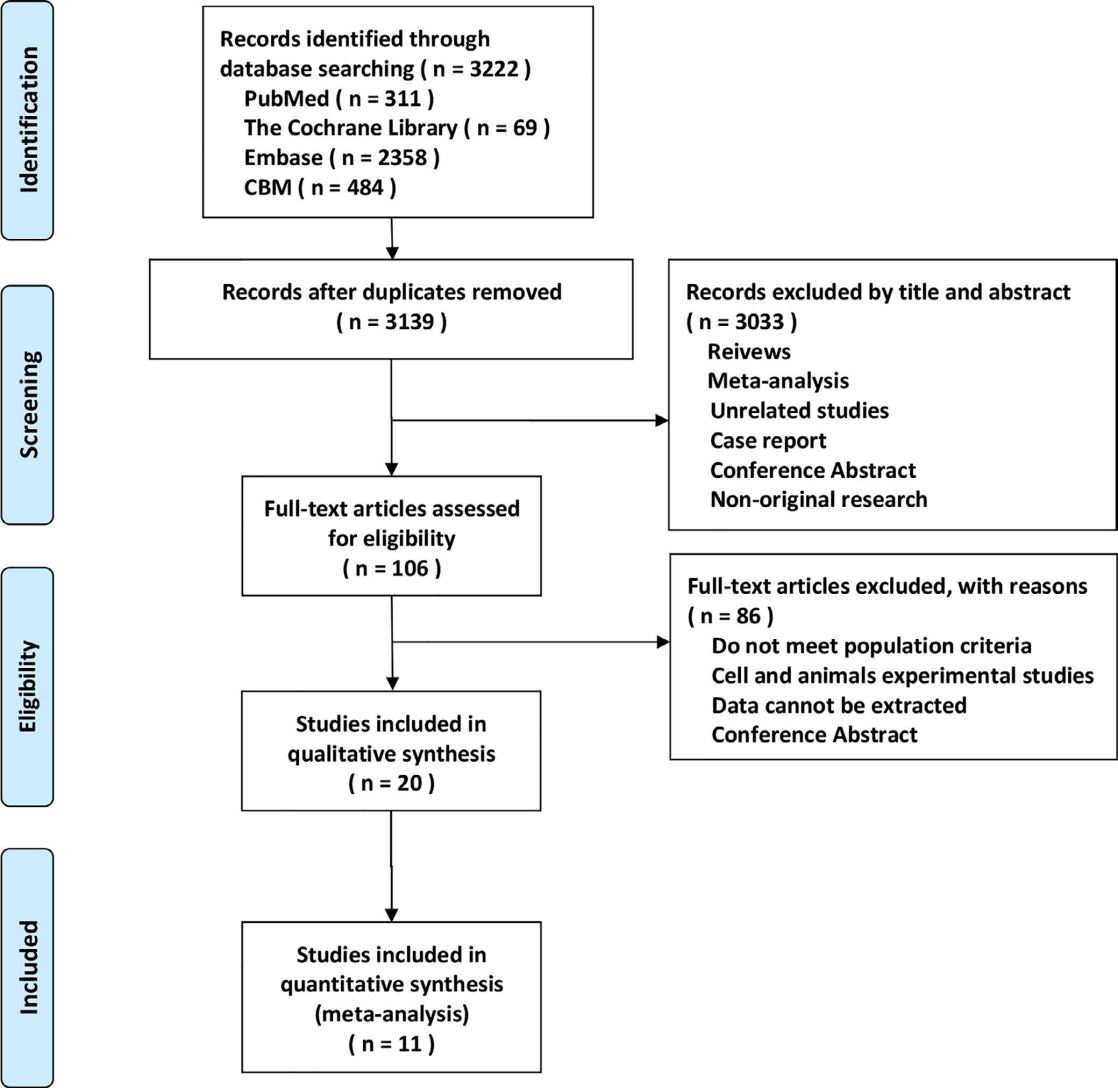
Flow diagram of the identification of eligible studies.

Two investigators independently searched for articles in the China Biology Medicine, EMBASE, Cochrane Library, and PubMed databases from inception until April 28, 2020, and no language restrictions were applied. We used the following search terms: “Carcinoma, Non‐Small‐Cell Lung,” “Programmed death‐1,” “Programmed death ligand‐1,” “Radiotherapy,” and “Chemoradiotherapy.” All relevant keyword variants for these terms were used. Figure 1 shows PRISMA flow diagram. Supplement 1 shows the detailed search strategy for each database.

### 2.2 Inclusion and exclusion criteria

The inclusion criteria were as follows: Original papers of human clinical trials that reported the outcomes of combination therapy using PD‐1/PD‐L1 inhibitors and RT in patients with NSCLC. There were no restrictions on tumor stage, publication date, study population, language, or study design.

The exclusion criteria were as follows: (i) Studies that reported NSCLC concomitant with other malignant neoplasms, (ii) studies from which data could not be extracted, (iii) duplicate reports (only the latest or the parent study was included), (iv) studies that reported only protocols, and (v) abstracts for which the complete text was unavailable.

### 2.3 Study selection

After the literature search, two investigators discarded all duplicate studies obtained from various databases and independently screened the titles and abstracts of all remaining articles to exclude manuscripts that did not meet the inclusion criteria. Furthermore, the investigators carefully checked the complete texts of articles that were deemed potentially eligible for inclusion in this research. Finally, a third investigator discussed the selections with the two investigators who performed the screening. Any disagreement regarding study eligibility that persisted after this discussion was resolved by consulting a fourth investigator.

### 2.4 Data extraction

The two aforementioned investigators independently extracted the following information from each trial: (i) the characteristics of the included trials, including the country, year of publication, author names, baseline characteristics, number of patients, smoking status, PD‐L1 expression status, oncogene driver mutation status, disease stage, PD‐1/PD‐L1 inhibitors and dose, RT dose (Gy)/fraction, the sequence of administration of PD‐1/PD‐L1 inhibitors and RT, RT target sites, RT techniques, and follow‐up duration. (ii) Long‐term outcomes represented by the OS and PFS. (iii) Short‐term outcomes represented by the objective response rate (ORR) and the disease control rate (DCR). (iv) AEs (regardless of grade).

### 2.5 Quality assessment

The two aforementioned investigators independently assessed the methodological quality of the randomized controlled trials (RCTs) using the Cochrane Handbook for Systematic Reviews of Interventions, version 5.1.0 (Cochrane Collaboration Risk of Bias Tool).^21^ The Newcastle‐Ottawa Scale was used for cohort or case‐control studies. The Case Series Report Quality Evaluation Form was used for single‐arm studies.^22^ The details of the quality assessment are display in Tables S1–S3 (Supplement 2).

### 2.6 Statistical analysis

We used the Stata software, version 12.0 (2011, Stata Corp, College Station, TX, USA) for data analyses. Odds ratios (ORs) and their 95% confidence intervals (CIs) were used as summary statistics for dichotomous data. A two‐tailed *p* value of 0.05 was considered statistically significant. Data were combined using a random‐effects model, and statistical heterogeneity was evaluated using the I^2^ test. A Higgins I^2^ statistic <50% represented low heterogeneity and >50% represented high heterogeneity. Subgroup analyses were performed to determine the source of heterogeneity and the factors associated with clinical benefits, if any.

## 3 RESULTS

### 3.1 Study characteristics

Approximately 2027 patients were enrolled in the 20 studies included in this research. With regard to the study design, nine studies were non‐randomized controlled trials (NRCT),^6^, ^7^, ^8^, ^23^, ^24^, ^25^, ^26^, ^27^, ^28^ nine were single‐arm studies,^29^, ^30^, ^31^, ^32^, ^33^, ^34^, ^35^, ^36^, ^37^ and two were RCTs.^5^, ^9^ All studies included at least one arm treated with combination therapy using PD‐1/PD‐L1 inhibitors and RT. Six studies compared the administration of RT plus PD‐1/PD‐L1 inhibitors with PD‐1/PD‐L1 inhibitors alone, and five studies compared the administration of RT plus PD‐1/PD‐L1 inhibitors with RT alone. Of the 20 studies, 13 reported chemotherapy regimens used and seven did not describe these regimens. Most chemotherapy regimens were platinum‐doublet or platinum‐based; however, the exact number of chemotherapy cycles administered was unknown. Table 1 summarizes the baseline characteristics of the included studies (the RT technique, RT dose, the sequence of PD‐1/PD‐L1 inhibitors and RT administered, and types of PD‐1/PD‐L1 inhibitors used). Table 2 summarizes the characteristics of patients included in the studies (age, sex, histopathological features, smoking status, PD‐L1 status, oncogene driver mutation status, Eastern Cooperative Oncology Group status, or the Karnofsky Performance Status). Tables S1–S3 (Supplement 2) show the results of the quality analysis.

**TABLE 1:** Characteristics of the included studies.

Author	Year	Nation	Study type	Group	N	Tumor stage	CT status, N (%)	RT target	RT technique	RT dose/fraction	ICIs sequencing	ICIs type
Shaverdian	2017	America	NRCT	RT+ICI	42	IV	Not clear	MRT	Con‐RT SRS SBRT	Not clear	Sequential (After RT)	Anti‐PD−1 Pembrolizumab
				ICI	55							
Tamiya	2017	Japan	NRCT	RT+ICI	50	IV	Not clear	TRT	Not clear	Not clear	Sequential (After RT)	Anti‐PD−1 Nivolumab
				ICI	151							
Fiorica	2018	Italy	NRCT	RT+ICI	15	IIIB‐IV	Prior CT≧ 1cycle platinum‐based	MRT	3DCRT IMRT SBRT	36 Gy/12F 8–16 Gy/1‐2F	Sequential (After RT)	Anti‐PD−1 Nivolumab
				ICI	20							
Theelen	2019	Netherland	RCT Phase II	RT+ICI	36	IV	Prior CT≧ 1cycle Regimen not clear	MRT	SBRT	24 Gy/3F	Sequential (After RT)	Anti‐PD−1 Pembrolizumab
				ICI	40							
Yamaguchi	2019	Japan	NRCT	RT+ICI	66	III‐IV	Prior CT ≧ 1cycle Most platinum‐based	MRT	Not clear	Bone 8–30 Gy Thorax 30–60 Gy Brain 30–50 Gy	Sequential (After RT)	Anti‐PD−1 Nivolumab
				ICI	58							
Ratnayake	2020	Australia	NRCT	RT+ICI	65	IV	Prior CT platinum doublet	MRT	Not clear	30 Gy (8–66 Gy) 20 Gy (6–50 Gy)	Sequential (After RT) Concurrent	Anti‐PD−1 Nivolumab
				ICI	20							
Hubbeling	2018	America	NRCT	RT+ICI	50	IV With BM	Prior CT platinum doublet	MRT	WBRT PBI SRS	Not clear WBRT and PBI 10‐15F SRS 1‐5F	Sequential (After and Before RT) Concurrent	Anti‐PD−1 Anti‐PD‐L1
				RT	113							
Shepard	2019	America	NRCT	RT+ICI	17	IV With BM	Prior CT Most platinum doublet	MRT	SRS	18.4 Gy±2.3 Gy	Concurrent	Nivolumab Pembrolizumab Atezolizumab
				RT	34					19.3 Gy±2.5 Gy		
Fukui	2020	Japan	NRCT	RT+ICI	18	IIIA‐IIIB	Prior CT ≧ 2cycle platinum‐based	TRT	3DCRT	60 (10–66)Gy	Sequential (After RT)	Nivolumab Pembrolizumab Atezolizumab
				RT	90							
Gray	2020	Multicenter	RCT Phase III	RT+ICI	476	III	Prior CT ≧ 2cycle platinum‐based	TRT	Not clear	<54 Gy ≥54 Gy to ≤66 Gy >66 Gy to ≤74 Gy	Sequential (After RT)	Anti‐PD‐L1 Durvalumab
				RT	237							
Singh	2020	America	NRCT	RT+ICI	39	IV With BM	Prior CT Regimen and cycle not clear	MRT	SRS	18 Gy (12–24 Gy)	Sequential Concurrent	Nivolumab Pembrolizumab Ipilimumab nivolumab Atezolimumab
				RT	46							
Ahmed	2017	America	NRCT Single arm	RT+ICI	17	IV With BM	Most prior CT Platinum‐based	MRT	SRS FSRT	18 Gy/1F, 20 Gy/1F, 21 Gy/1F, 24 Gy/1F, 25 Gy/5F	Sequential Concurrent	Anti‐PD−1 Anti‐PD‐L1
Lesueur	2018	France	NRCT Single arm	RT+ICI	104	IV	Not clear	MRT	3DCRT SRS IMRT Other	30 Gy (29.6–30.0 Gy)	Sequential Concurrent	Anti‐PD−1 Nivolumab
Schapira	2018	America	NRCT Single arm	RT+ICI	37	IV With BM	Prior CT Regimen and cycle not clear	MRT	SRS	25 Gy/5F, 22 Gy/2F, 21 Gy/3F, 20 Gy/2F, 18 Gy/1F, 17 Gy/1F, 16 Gy/1F, 15 Gy/1F	Sequential Concurrent	Nivolumab Pembrolizumab Atezolizumab
Miyamoto	2019	Japan	NRCT Single arm	RT+ICI	6	IV	Prior CT Regimen and cycle not clear	MRT	SRT	33 Gy/3F, 30 Gy/3F, 38 Gy/4F, 48 Gy/4F, 25.5 Gy/3F	Sequential (After RT)	Anti‐PD−1 Nivolumab
Qin	2019	America	NRCT Single arm	RT+ICI	12	IV	Prior CT platinum doublet	MRT	HIGRT	12–50 Gy, 6–8 Gy/F, 3F−5F	Concurrent	Anti‐PD‐L1 Atezolizumab
Amino	2020	Japan	NRCT Single arm	RT+ICI	20	III	Prior CT platinum doublet	TRT	Not clear	54–60 Gy	Sequential (After RT)	Anti‐PD−1 Nivolumab Pembrolizumab
Chu	2020	China	NRCT Single arm	RT+ICI	31	III	Prior CT platinum doublet	TRT	Not clear	66–70 Gy/32‐35F 60–66 Gy >66 Gy	Sequential (After RT)	Anti‐PD‐L1 Durvalumab
Jabbour	2020	America	NRCT Single arm	RT+ICI	21	III	Prior CT Platinum doublet	TRT	IMRT VMAT Proton	60 Gy 40 Gy	Concurrent	Anti‐PD−1 Pembrolizumab
Miura	2020	Japan	NRCT Single arm	RT+ICI	41	III	Prior CT platinum‐based	TRT	4D‐CT IGRT	60 Gy/30F 54 Gy/25F	Sequential (After RT)	Anti‐PD‐L1 Durvalumab

BM, brain metastasis; CT, chemotherapy; RT, radiotherapy; ICIs, immune checkpoint inhibitors; TRT, thoracic radiotherapy; MRT, radiotherapy of metastases; Co‐RT, conventional radiotherapy; 3DCRT, 3‐dimensional‐conformal radiation therapy; IMRT, intensity modulated radiation therapy; SRS, stereotatic radiosurgery; SBRT, stereotactic body radiation therapy; PBI, partial brain irradiation; FSRT, fractionated stereotactic radiotherapy; HIGRT, hypofractionated image‐guided radiation therapy; VMAT, volumetric modulated arc therapy; IGRT, image‐guided radiotherapy; IMRT, intensity modulated radiation therapy.

**TABLE 2:** Patients characteristics of the included studies.

Author	Year	Group	N	Age median (range)	SEX, n (%)		Histology, n (%)			ECOG, n (%)		Smoking status, n (%)		PD‐L1 status	Oncogene driver mutation		
					Male	Female	Sq	Ad	Oth	0–1	≧2	Yes	No		EGFR	KRAS	Oth
Shaverdian	2017	RT+ICI	42	65 (36.0–77.0)	21 (50.0)	21 (50.0)	11 (20.0)	31 (74.0)		42 (100.0)	0	23 (55.0)	19 (45.0)	P 30 (71.0) N 5 (12.0) U 7 (17.0)	‐		
		ICI	55	66 (32.0–83.0)	29 (53.0)	26 (47.0)	8 (15.0)	47 (85.0)		55 (100.0)	0	30 (55.0)	25 (45.0)	P 44 (80.0) N 6 (11.0) U 5 (9.0)	‐		
Tamiya	2017	RT+ICI	50	68 (27–87)	135 (67.2)	66 (32.8)	42 (20.9)	142 (70.6)	17 (8.5)	153 (76.1)	48 (23.9)	44 (21.9)	157 (78.1)	‐	P 36 (17.9)/N 128 (63.7) U 37 (18.4)		
		ICI	151	68 (27–87)													
Fiorica	2018	RT+ICI	15	70 (44–81)	11 (73.0)	4 (27.0)	9 (60.0)	6 (40.0)		9 (60.0)	6 (40.0)	14 (93.0)	1 (7.0)	‐	‐		
		ICI	20	69 (53–77)	19 (95.0)	1 (0.5)	10 (50.0)	10 (50.0)		14 (70.0)	6 (30.0)	18 (90.0)	2 (10.0)				
Theelen	2019	RT+ICI	36	62 (35–78)	20 (55.6)	16 (44.4)	5 (13.9)	31 (86.1)		36 (100.0)	0	36 (100.0)	0	0: 18 (50.0) 1–49%: 8 (22.2) ≥50%: 10 (27.8)	‐		
		ICI	40		23 (57.5)	17 (42.5)	4 (10.0)	36 (90.0)		40 (100.0)	0	40 (100.0)	0	0: 25 (62.5) 1–49%:8 (20.0) ≥50%: 5 (12.5)			
Yamaguchi	2019	RT+ICI	66	69 (31–85)	51 (77.3)	15 (22.7)	35 (53.0)	31 (47.0)		0: 31 (47.0)	1–3: 35 (53.0)	52 (78.8)	14 (21.2)	‐	P 10 (15.2)/N 51 (77.3)		
		ICI	58		42 (72.4)	16 (27.6)	24 (41.4)	34 (58.6)		0: 18 (31.0)	1–3: 40 (69.0)	47 (81.0)	11 (19.0)		P 4 (6.9)/N 53 (91.4)		
Ratnayake	2020	RT+ICI	65	65 (42–84)	44 (67.7)	21 (32.3)	20 (30.8)	41 (63.1)	4 (6.2)	40 (61.5)	23 (35.4)	58 (89.2)	7 (10.8)	‐	‐		
		ICI	20		9 (45.0)	11 (55.0)	8 (40.0)	12 (60.0)	0	13 (65.0)	7 (35.0)	17 (85.0)	3 (15.0)				
Hubbeling	2018	RT+ICI	50	61 (35–82)	16 (32.0)	34 (68.0)	8 (16.0)	38 (76.0)	4 (8.0)	M(R):1 (0–3)		‐		‐	‐		
		RT	113	62 (31–97)	42 (37.0)	71 (63.0)	11 (10.0)	99 (88.0	3 (3.0)	M(R):1 (0–4)							
Shepard	2019	RT+ICI	17	64.4 ± 8.6	11 (64.7)	6 (35.3)	‐			‐		‐		‐	‐		
		RT	34	64.1 ± 10.2	20 (58.8)	14 (41.2)											
Fukui	2020	RT+ICI	18	65 (36–76)	81 (75.0)	27 (25.0)	38 (35.0)	46 (43.0)	24 (22.0)	106 (98.0)	2 (2.0)	97 (90.0)	11 (10.0)	‐	P 2 (2.0)/N 69 (62.0) U 32 (30.0)	‐	ALK 4 (4.0) ROS1 1 (1.0)
		RT	90														
Gray	2020	RT+ICI	476	64 (31–84)	334 (70.2)	142 (29.8)	224 (47.1)	252 (52.9)		476 (100.0)	‐	433 (91.0)	43 (9.0)	≥25%: 115 (24.2) <25%: 187 (39.3)	P 29 (6.1)/N 315 (66.2) U 132 (27.7)		
		RT	237	64 (23–90)	166 (70.0)	71 (30.0)	102 (43.0)	135 (57.0)		237 (100.0)	‐	216 (91.1)	21 (8.9)	≥25%: 44 (18.6) <25%: 105 (44.3)	P 14 (5.9)/N 165 (69.6) U 58 (24.5)		
Singh	2020	RT+ICI	39	61.9 (28–87.5)	12 (30.7)	23 (50.0)	6 (15.0)	28 (72.5)	‐	KPS:80 (50–100)		‐		P 39 (100.0)	‐		
		RT	46	62.5 (31.8–79.4)	27 (69.2)	23 (50.0)	3 (6.5)	29 (63.0)	‐	KPS:90 (60–100)				‐			
Ahmed	2017	RT+ICI	17	60 (44–79)	10 (58.8)	7 (41.2)	‐			KPS:90: 9 (53.0) 80: 5 (29.0) 70: 3 (18.0)		‐		‐	2 (12.0)	3 (18.0)	Both EGFR and KRAS:1 (6.0)
Lesueur	2018	RT+ICI	104	60.3 (54.5–67.1)	67 (64.4)	37 (35.6)	65 (62.5)	34 (32.7)	5 (4.8)	69 (66.4)	35 (33.5)	96 (92.3)	8 (7.7)	‐	2 (1.9)	22 (21.2)	ALK:2 (1.9) MET:5 (4.8) Others:3 (2.9)
Schapira	2018	RT+ICI	37	63 (42–84)	13 (35.1)	24 (64.9)	‐			KPS:90–100:24 (64.9) 70–80:12 (32.4) 60:1 (2.7)		‐		‐	‐		
Miyamoto	2019	RT+ICI	6	58 (45–72)	4 (66.7)	2 (33.3)	‐	5 (83.3)	1 (16.7)	0–2: 6 (100.0)		4 (66.7)	2 (33.3)	≥50%:4 (66.7)	2 (33.3)	6 (100.0)	‐
Qin	2019	RT+ICI	12	64 (55–77)	8 (66.7)	4 (33.3)	2 (16.7)	7 (58.3)	3 (25.0)	0–2: 12 (100.0)		1 (91.7)	1 (8.3)	P: 9 (75.0) U: 3 (25.0)	‐		
Amino	2020	RT+ICI	20	59 (42–73)	18 (90.0)	2 (10.0)	14 (70.0)	‐	6 (30.0)	0: 8 (40.0) 1: 11 (55.0) 2: 1 (5.0)		19 (95.0)	1 (5.0)	<1%: 1 (5.0) 1–49%: 4 (20.0) ≥50%: 5 (25.0) NE: 10 (50.0)	‐		
Chu	2020	RT+ICI	31	64 (52–74)	26 (83.9)	5 (16.1)	8 (25.8)	20 (64.5)	3 (9.7)	0: 25 (80.7) 1: 5 (16.1) 2: 1 (3.2)		23 (74.2)	8 (25.8)	P: 14 (45.2) N: 6 (19.3) U: 11 (35.5)	4 (12.9)	‐	ALK:1 (3.3)
Jabbour	2020	RT+ICI	21	69.5 (53–85)	10 (48.0)	11 (52.0)	10 (48.0)*	11 (52.0)		21 (100.0)	‐	20 (95.0)	1 (5.0)	<1%: 4 (21.0) 1–49%: 10 (53.0) ≥50%: 5 (26.0)	‐		
Miura	2020	RT+ICI	41	72 (51–80)	33 (80.0)	8 (20.0)	15 (37.0)	21 (51.0)	5 (12.0)	0: 24 (58.0) 1: 17 (42.0)	‐	33 (80.0)	8 (20.0)	<1%: 12 (29.0) 1–49%: 11 (27.0) ≥50%: 9 (22.0) NE: 9 (22.0)	5 (12.0)		ROS1:1 (3.0) Unknown:35 (85.0)

RT, radiotherapy; ICI, immune checkpoint inhibitors; Sq, squamous; Ad, adenocarcinoma; Oth, other; ECOG, Eastern Cooperative Oncology Group; M(R), median(range); P, positive; N, negative; U, unknown; KPS, Karnofsky; NE, not evaluate.

### 3.2 Long‐term efficacy outcomes: Overall survival and progression‐free survival

**FIGURE 2:**
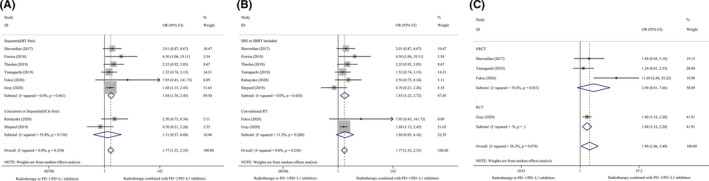
Subgroup analyses of 1–2 year OS. (A), 1‐year OS, Sequence of RT and ICIs. (B), 1‐year OS, RT techniques. (C), 2‐year OS, Study design. RT, radiotherapy; ICIs, immune checkpoint inhibitors; OS, overall survival; SBRT, stereotactic body radiotherapy; SRS, stereotactic radiosurgery; RCT, randomized controlled trial; NRCT, non‐randomized controlled trial.

Survival outcomes were the primary endpoints in our systematic review and meta‐analysis. Eight studies^5^, ^6^, ^7^, ^8^, ^9^, ^24^, ^25^, ^26^ were included in this meta‐analysis. Compared with non‐combination therapy, combination radio‐immunotherapy using PD‐1/PD‐L1 inhibitors and RT was significantly associated with longer 1‐year OS (I^2^ = 0.0%, OR 1.77, 95% CI 1.35–2.33, *p* = 0.000) and 2‐year OS (I^2^ = 56.3%, OR 1.77, 95% CI 1.35–2.33, *p* = 0.000) in patients with advanced NSCLC. Subgroup analysis based on the study design (RCT or NRCT) showed differences in 2‐year OS (RCT: OR 1.60, 95% CI 1.16–2.20, *p* = 0.004, vs NRCT: I^2^ = 70.6%, OR 2.50, 95% CI 0.81–7.66, *p* = 0.110). We also investigated the effect of the sequence of treatment rendered and the RT techniques. Subgroup analyses showed longer survival time in the sequential RT‐first group than in the concurrent or sequential ICI‐first group. With regard to RT techniques, combination therapy using SBRT or SRS and PD‐1/PD‐L1 inhibitors was associated with better survival benefits than combination therapy using conventional RT and PD‐1/PD‐L1 inhibitors. Figure 2 shows the results of the meta‐analysis and subgroup analyses of the 1‐ and 2‐year OS rates depicted as forest plots.

**FIGURE 3:**
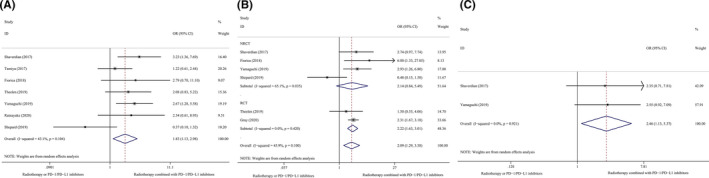
Subgroup analyses of 0.5–2 year PFS. (A), 0.5‐year PFS, Overall population. (B), 1‐year PFS, Study design. (C), 2‐year PFS, Overall population. PFS, progression‐free survival; RCT, randomized controlled trial; NRCT, non‐randomized controlled trial.

Compared with non‐combination therapy, combination radio‐immunotherapy using PD‐1/PD‐L1 inhibitors and RT was significantly associated with a longer 0.5‐year PFS (I^2^ = 43.1%, OR 1.83, 95% CI 1.13–2.98, *p* = 0.014), 1‐year PFS (I^2^ = 45.9%, OR 2.09, 95% CI 1.29–3.38, *p* = 0.003), and 2‐year PFS (I^2^ = 0.0%, OR 2.47, 95% CI 1.13–5.37, *p* = 0.023) in patients with advanced NSCLC. Subgroup analysis based on the study design (RCT or NRCT) also showed differences in the 1‐year PFS (RCT: I^2^ = 0.0%, OR 2.22, 95% CI 1.63–3.01, *p* = 0.000 and NRCT: I^2^ = 65.1%, OR 2.14, 95% CI 0.84–5.49, *p* = 0.112). Figure 3 shows the results of the meta‐analysis and subgroup analyses of the 0.5‐, 1‐, and 2‐year PFS depicted as forest plots.

Among the eight single‐arm studies that investigated patients who received combination therapy using PD‐1/PD‐L1 and RT, the 0.5‐year OS was 88.1% (median) (range 81.0%–95.2%), the 1‐year OS was 50.5% (48.8%–85.2%), and the 2‐year OS was 27.1% (25.0%–29.1%). The median OS duration was 21.6 months (6.9–29.4 months). Only one study reported a 0.5‐year PFS rate of 81.0%. The 1‐year PFS was 42.5% (20.9%–67.9%), and only one study reported 2‐year PFS of 10.1%. The median PFS was 8.4 months (2.3–18.7 months) (Table 3).

**TABLE 3:** Outcomes of single‐arm studies.

Author	Year	N	ORR (%)	DCR (%)	6‐month OS (%)	1‐year OS (%)	2‐year OS (%)	6‐month PFS (%)	1‐year PFS (%)	2‐year PFS (%)	Median OS (month)	Median PFS (month)	AE<G3 (%)	AE≧G3 (%)	*p*<G3 (%)	P≧G3 (%)
Ahmed	2017	17	‐	‐	81.0	51.0	‐	‐	‐	‐	17.9	‐	‐	11.8	‐	‐
Lesueur	2018	104	‐	‐	‐	48.8	29.1	‐	20.9	10.1	25.2	2.7	85.6	14.4	3.3	1.1
Schapira	2018	37	‐	‐	‐	‐	‐	‐	‐	‐	‐	‐	‐	‐	‐	‐
Miyamoto	2019	6	50.0	66.7	‐	‐	‐	‐	‐	‐	‐	4.6	‐	16.7	0	16.7
Qin	2019	12	25.0	50.0	‐	‐	‐	‐	‐	‐	6.9	2.3	8.3	41.7	0	8.3
Amino	2020	20	45.0	65.0	‐	50.0	29.1	‐	28.5	‐	26.2	8.4	30.0	5.0	5.0	0
Chu	2020	31	25.8	80.6	‐	‐	‐	‐	56.4	‐		12	58.6	13.8	10.3	6.9
Jabbour	2020	21	‐	‐	95.2	85.2	‐	81.0	69.7	‐	29.4	18.7	‐	‐	28.0	5.0
Miura	2020	41	‐	‐	‐	‐	‐	‐	‐	‐	15.4	14.1	‐	‐	58.5	2.4

ORR, objective response rate; DCR, disease control rate; OS, overall survival; PFS, progression‐free survival; AE, adverse events; P, pneumonitis.

### 3.3 Short‐term efficacy outcomes: Evaluation of response to treatment

The tumor response rate, which was determined using the Response Evaluation Criteria in Solid Tumors guideline, version 1.1 was the secondary endpoint in this meta‐analysis. One study used the Immune‐related Response Criteria^6^ and another used the Response Assessment in Neuro‐oncology tool.^26^ Results of the meta‐analysis showed that combination therapy using PD‐1/PD‐L1 inhibitors and RT improved the ORR (I^2^ = 78.1%, OR 2.76, 95% CI 1.06–7.19, *p* = 0.038) and DCR (I^2^ = 0.0%, OR 1.80, 95% CI 1.21–2.68, *p* = 0.004).

**FIGURE 4:**
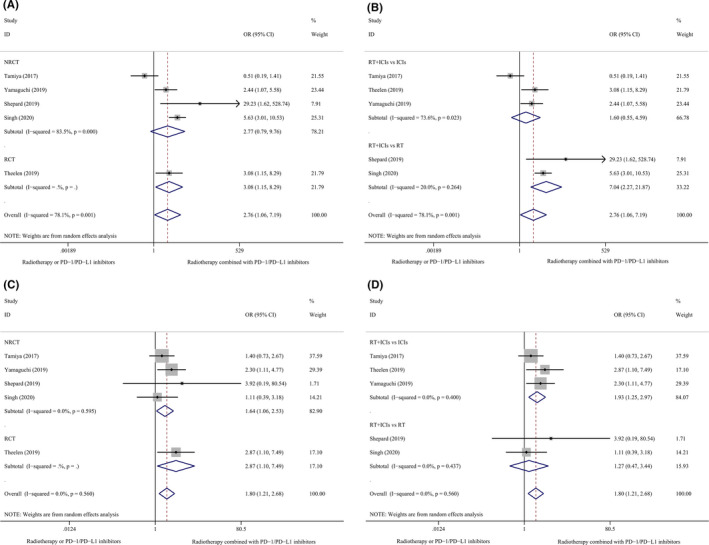
Subgroup analyses of ORR and DCR. (A), ORR, Study design. (B), ORR, Different groups. (C), DCR, Study design. (D), DCR, Different groups. RT, radiotherapy; ICIs, immune checkpoint inhibitors; ORR, objective response rate; DCR, disease control rate; RCT, randomized controlled trial; NRCT, non‐randomized controlled trial.

Subgroup analyses based on the study design (RCT vs NRCT) showed differences in ORRs (RCT: OR 3.08, 95% CI 1.15–8.29, *p* = 0.026 vs NRCT: I^2^ = 83.5%, OR 2.77, 95% CI 0.79–9.76, *p* = 0.112). However, the DCR did not significantly differ (RCT: OR 2.87, 95% CI 1.10–7.49, *p* = 0.031 vs NRCT: I^2^ = 0.0%, OR 1.64, 95% CI 1.06–2.53, *p* = 0.026). Additionally, this meta‐analysis showed that the DCRs were significantly higher in patients who received combination therapy using PD‐1/PD‐L1 inhibitors and RT than in patients who received ICIs alone (I^2^ = 0.0%, OR 1.93, 95% CI 1.25–2.97, *p* = 0.003); however, no significant differences were observed in the ORRs. Moreover, the ORRs were significantly higher in patients who received combination therapy using PD‐1/PD‐L1 inhibitors and RT than in patients who received RT alone (I^2^ = 20.0%, OR 7.04, 95% CI 2.27–21.87, *p* = 0.001); however, no significant differences were observed in the DCRs. The median ORR and DCR were 35.4% (25.0%–50.0%) and 65.9% (50.0%–80.6%), respectively in single‐arm studies (Table 3). Figure 4 shows the results of the meta‐analysis and subgroup analyses of the ORR and DCR depicted as forest plots.

### 3.4 Adverse events

AEs were defined using the National Cancer Institute‐Common Terminology Criteria for Adverse Events (NCI‐CTCAE), version 4.0. One study used the NCI‐CTCAE, version 5.0^31^ and another used the NCI‐CTCAE, version 3.0.^7^ The included studies primarily reported the total AE rates and immune‐related or radiation pneumonitis rates associated with combination therapy using PD‐1/PD‐L1 inhibitors and RT. The AEs described included fatigue, dermatitis, skin rash, diarrhea, nausea, constipation, anemia, neutropenia, leukopenia, thrombocytopenia, hypothyroidism, and abnormal liver function.

Results of the meta‐analysis showed that combination therapy using PD‐1/PD‐L1 inhibitors and RT did not increase the serious AE rates (≥grade 3) (I^2^ = 0.0%, OR 1.24, 95% CI 0.88–1.74, *p* = 0.222) or the mild AE rates (grades 1 and 2) (I^2^ = 0.0%, OR 0.97, 95% CI 0.71–1.34, *p* = 0.858) compared with administration of PD‐1/PD‐L1 inhibitors or RT rendered alone. Notably, combination therapy using PD‐1/PD‐L1 inhibitors and RT was not associated with a high risk of serious (≥grade 3) immune‐related or radiation pneumonitis, although rates of mild (grade 1–2) pneumonitis were increased in patients who received combination therapy. Subgroup analyses revealed that thoracic RT (OR 1.47, 95% CI 1.02–2.12, *p* = 0.040) was associated with a higher incidence of grade 1–2 pneumonitis than RT administered to metastatic lesions (I^2^ = 0.0%, OR 5.09, 95% CI 0.86–30.24, *p* = 0.074, Cross *p* = 0.180). The risk of grade 1–2 pneumonitis was higher in those who received PD‐L1 inhibitors (OR 1.47, 95% CI 1.02–2.12, *p* = 0.040) than in those who received PD‐1 inhibitors (I^2^ = 0.0%, OR 5.09, 95% CI 0.86–30.24, *p* = 0.074, Cross *p* = 0.180).

**FIGURE 5:**
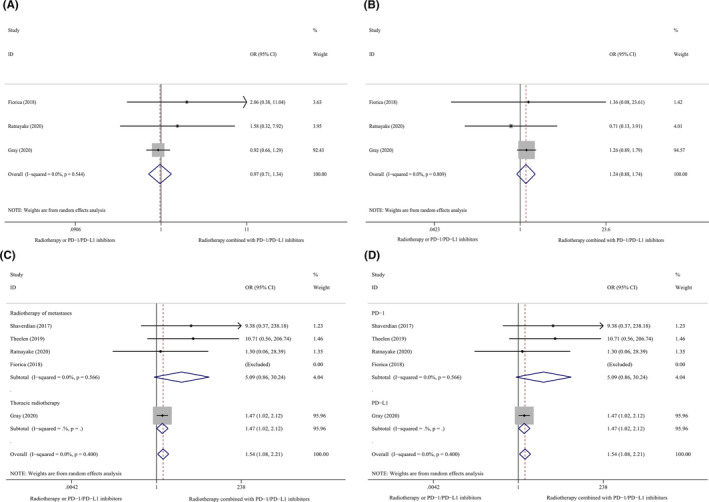
Subgroup analyses of AEs and mild pneumonitis. (A), AEs ≥grade 3, Overall population. (B), AEs grade 1–2, Overall population. (C), Pneumonitis grade 1–2, The site of RT. (D), Pneumonitis grade 1–2, PD‐1 vs PDL1. RT, radiotherapy; AEs, adverse events.

Three studies that reported combination therapy using SRS and PD‐1/PD‐L1 inhibitors^23^, ^26^, ^27^ compared the incidence of cerebral radiation‐induced necrosis in patients with brain metastases. Notably, combination therapy using PD‐1/PD‐L1 inhibitors and RT did not significantly increase the incidence of cerebral radiation‐induced necrosis One study showed no significant differences in the progression of brain peritumoral edema between patients with NSCLC treated with and without combination therapy.^26^ The median incidence rate of mild AEs was 44.3% (8.3%–85.6%), and the median incidence rate of serious AEs was 14.1% (5.0%–41.7%) in single‐arm studies included in our research. The incidence rate of mild pneumonitis was 5% (0.0%–58.5.0%), and the median incidence rate of serious pneumonitis was 5% (0.0%–16.7%) (Table 3). Figure 5 shows the results of the meta‐analysis and subgroup analyses of the AEs and mild pneumonitis depicted as forest plots.

## 4 DISCUSSION

Our results suggest that combination therapy using PD‐1/PD‐L1 inhibitors and RT may improve OS, PFS, and tumor response rates in patients with advanced NSCLC. Moreover this therapeutic approach did not significantly increase the risk of serious AEs.

We observed that combination therapy using PD‐1/PD‐L1 inhibitors and RT was associated with prolonged 1‐ and 2‐year OS, improved 0.5, 1‐, and 2‐year PFS, and improved ORRs and DCRs. The higher survival benefit and better tumor response rates associated with combined radio‐immunotherapy could be attributed to the following mechanisms: The PD‐1 receptor is highly expressed on activated infiltrating T cells induction by the tumor microenvironment, and PD‐L1 is expressed on the surface of tumor cells. Tumor cells can trigger the PD‐1 pathway to escape the anti‐tumor immune response, and PD‐1 or PD‐L1 inhibitors can restore T‐cell signaling, with subsequent reactivation of the antitumor activity of specific CD8+T cells. Recruitment and activation of CD8+T cells are the key processes involved in the therapeutic actions of PD‐1/PD‐L1 inhibitors.^3^ (i) RT induces and enhances the immunogenicity of tumors by increasing the expression of tumor‐associated antigens, the major histocompatibility complex, and damage‐associated molecular patterns.^38^ CD8+T and dendritic cells subsequently recruit and activate accelerately, inducing the anti‐tumor immunological effect in the body.^38^, ^39^, ^40^, ^41^ (ii) RT also regulates the tumor microenvironment, increases the levels of chemokines CXCL10 and CXCL16 in the tumor microenvironment, and promotes the migration of CD8+T cells to the tumor site.^42^ (iii) RT activates the cGAS/STING pathway to produce an effective immune response.^43^ (iv) ICIs not only activate CD8+T cells but also normalize the tumor vasculature, reduce the state of tumor hypoxia, and increase tumor radiosensitivity.^44^ (v) “Abscopal effects” have been detected at distant tumor sites that do not receive radiation during RT. Therefore, combination therapy using PD‐1/PD‐L1 inhibitors and RT may promote “abscopal effects” and enhance the effectiveness of cancer immunotherapy.^45^ The aforementioned mechanisms could contribute to the short‐ and long‐term benefits of combination therapy using PD‐1/PD‐L1 inhibitors and RT in patients with advanced NSCLC.

The sequence of administration of combination therapy using PD‐1/PD‐L1 inhibitors and RT is the focus of investigation in this field; however, currently, the optimal sequence remains unclear. Subgroup analyses performed in our study showed that PD‐1/PD‐L1 inhibitor administration following RT outperformed in which concurrent PD‐1/PD‐L1 inhibitor and RT followed PD‐1/PD‐L1 inhibitor therapy. This result is consistent with the theory established by preclinical studies, which proposes that RT upregulates PD‐L1 expression through DNA double‐strand breaks and increased CD8+T cell infiltration.^46^, ^47^ Higher PD‐L1 expression was shown to improve the efficacy of PD‐1/PD‐L1 inhibitors and increase CD8+T cell infiltration. Similar findings have also been reported in association with other diseases and particle therapy for cancer treatment. A study performed by Iijima et al.^48^ reported that carbon ion radiotherapy (CIRT) can upregulate PD‐L1 expression in adenocarcinoma of the uterine cervix. Moreover PD‐L1 expression in adenocarcinoma of the uterine cervix after CIRT was favorably associated with PFS. However, the time interval between RT and ICI administration should not be prolonged. The PACIFIC study showed that the OS benefit was significantly greater in patients who were administered immunotherapy within 14 days after concurrent chemoradiotherapy than in patients who were administered immunotherapy after 14–42 days.^5^

Subgroup analyses also revealed that combination therapy using SBRT or SRS and PD‐1/PD‐L1 inhibitors may be more effective than combination therapy using conventional RT and PD‐1/PD‐L1 inhibitors in patients with advanced NSCLC. The PEMBRO‐RT (a phase II RCT) reported that the long‐term OS benefit associated with SBRT was observed only in the PD‐L1‐negative subgroup,^9^ which indicates that RT, particularly SBRT, can alter PD‐L1 expression, thereby improving the efficacy of ICIs. The combination of immunotherapy and stereotactic ablative radiotherapy is referred to as ISABR.^49^ Notably, in addition to activation of the immune system, RT may cause lymphopenia, which consequently affects the efficacy of ICI therapy. Reportedly, the main factors that affect radiation‐induced lymphopenia are multicourse RT, multisite irradiation, high‐dose RT, and variations in the RT techniques utilized (SBRT tends to reduce this risk^10^). The favorable results of combination therapy using SBRT or SRS and PD‐1/PD‐L1 inhibitors can be attributed to the aforementioned advantage offered by SBRT. SBRT and particle beam radiotherapy would be useful to reduce the volume of normal tissue irradiation and also activate the immune system and would therefore be a better radiotherapeutic choice when combined with PD‐1/PD‐L1 inhibitors. Preclinical studies have investigated the effects of low‐dose irradiation or precision radiotherapy on the immune system,^50^, ^51^ and this subject will be the focus of future research.

Our results prove that combination therapy using PD‐1/PD‐L1 inhibitors and RT did not increase the rate of serious AEs (≥grade 3), although rates of mild (grade 1–2) pneumonitis were higher in patients who received combination therapy comprising PD‐1/PD‐L1 inhibitors and RT. Radiation or immune pneumonitis associated with combination therapy using RT and ICIs, particularly thoracic RT combined with ICIs, has attracted particular attention. Our subgroup analyses revealed that the incidence of pneumonitis was higher in those who underwent thoracic RT than in those who received RT for metastatic lesions. Previous studies have reported that the higher incidence rate of pneumonitis may be associated with the race, type of histopathological presentation, performance score (PS), and dose‐volume indices of the lungs. Multivariate analysis of the data obtained in the PACIFIC study showed that among the patients treated with durvalumab, Asian patients with non‐squamous tumors and poor PS were more likely to develop pneumonitis.^52^ With regard to patients treated with chemoradiotherapy combined with ICIs, the management of lung volume receiving ≥20 Gy (V20) may appear relatively more important; however, it is unclear whether further randomized studies with larger sample sizes are necessary to investigate low‐dose effects such as V5 and V10 on the lungs.^29^ Our study results concur with those of other studies; mild pneumonitis did not affect the primary outcome such as OS. Exploratory analysis of the PACIFIC study reported at the 2019 European Society for Medical Oncology (ESMO) meeting^53^ showed pneumonitis did not affect treatment completion with durvalumab. No significant difference was observed in the percentage of patients who completed 12‐month treatment with durvalumab between patients with or without pneumonitis (49.5% vs 48.1%), and the incidence of grade 3–4 AEs and AE‐induced mortality rates were not associated with pneumonitis in patients treated with durvalumab. Moreover, although pneumonitis was more common in patients in the durvalumab group, the mortality rate of patients with pneumonitis in the durvalumab group was lower than that of patients with pneumonitis in the placebo group, and pneumonitis did not affect the survival benefit conferred by consolidation therapy using durvalumab. AEs associated with combination therapy using RT and ICIs reported by the U.S. Food and Drug Administration were discussed at the 2020 American Society of Clinical Oncology (ASCO) meeting.^54^ Analysis of pooled data from 66 prospective trials of ICIs (which included 25,836 patients) showed that the incidence of hematological toxicity and pneumonitis associated with combined radio‐immunotherapy may be slightly higher than that of RT alone, whereas the incidence of colitis, hepatitis, and myocarditis may be slightly lower, although these differences were statistically nonsignificant. We observed that some of the included single‐arm studies reported a relatively high incidence of AEs or mild pneumonitis.^29^, ^31^, ^33^, ^36^ The method of administration of combined radio‐immunotherapy^33^ and the small sample size of some of the included studies (which is likely to have introduced a risk of bias) could have contributed to these results. Additionally, the AEs observed in the included single‐arm studies were similar to those in the PACIFIC study. It is reasonable to conclude that the safety profile of combination therapy using PD‐1/PD‐L1 inhibitors and RT is satisfactory.

Interestingly, multiple studies have suggested that multisystem immune‐related adverse events (iRAEs), such as immune pneumonitis, thyroiditis, and dermatitis, may prolong the PFS and OS or increase the ORR and reduce the progressive disease rate in patients with NSCLC treated with ICIs,^28^, ^33^, ^36^, ^55^, ^56^, ^57^ which suggests that iRAEs may serve as a marker of immune system activation. The role of multisystem iRAEs as possible predictive markers of the response to ICIs should be investigated in future studies.^58^ Our results also show this tendency. Although we observed a high incidence of mild pneumonitis, the long‐term survival and tumor response rates were significantly improved. However, achieving a balance between safety and efficacy of combined radio‐immunotherapy is a concern that should be addressed by future research.

Our subgroup analyses revealed that the incidence of mild pneumonitis was higher in patients who received PD‐L1 inhibitors than in those who received PD‐1 inhibitors; however, no significant intergroup difference was observed (Cross *p* = 0.180). Two meta‐analyses performed in patients with NSCLC showed a higher incidence of pneumonitis in patients treated with PD‐1 inhibitors than in those treated with PD‐L1 inhibitors.^59^, ^60^ A possible mechanism that could explain this finding is the fact that PD‐1 inhibitors can block PD‐1 binding to PD‐L2 leading to increased PD‐L2 binding to the receptor repulsive guidance molecule b, resulting in local T‐cell clonal expansion, which breaks the balance of respiratory tolerance and consequently increases the risk of pneumonitis. In our view, this difference in outcomes may be secondary to the design limitations of NRCTs^21^ (some of the included studies were small‐size studies) and the differences in treatment modalities, which may introduce an element of bias. No AEs were observed in the sequence/concurrent subgroup or the SBRT/conventional RT subgroup.

The 2020 ASCO meeting also reported the efficacy of combination therapy using RT and pembrolizumab in patients with metastatic NSCLC. The results of the pooled analysis of two randomized studies suggested that RT combined with ICIs could significantly improve the ORRs of lesions in the radiation field and significantly improve PFS. Furthermore, the partial remission rate was significantly higher in patients with metastatic NSCLC treated with SBRT than in patients treated with conventional RT^61^; this finding was similar to our results. Several clinical trials of radiochemotherapy combined with ICIs are ongoing^62^; the complete texts of these studies are unavailable and were not included in this meta‐analysis. We will update this systematic review and meta‐analysis in the future.

Following are the strengths of this systematic review and meta‐analysis: (a) We analyzed the sequence of administration of RT and PD‐1/PD‐L1 inhibitors and observed that RT administered before PD‐1/PD‐L1 inhibitors may be beneficial and that this approach could be useful in real‐world clinical practice in the future. (b) We investigated the effect of RT techniques such as SBRT or SRS; in our opinion, improvements in RT techniques and precision are important factors that would determine the efficacy of combination therapy. Knowledge regarding the aforementioned advantages of SBRT or SRS might change the RT dose fractionation in combination therapy using RT and ICIs. (c) To our knowledge, this is the first study to assess the efficacy and safety profile of combination therapy using PD‐1/PD‐L1 inhibitors and RT in patients with NSCLC.

Following are the limitations of this study: (a) The study included only 20 relevant RCTs; the small sample size is a drawback of this research. (b) We selected both RCTs and NRCTs in this study. Although subgroup analyses were performed, we were unable to eliminate the effects of the NRCT design on the results. (c) Most studies did not use blinding or random allocation. (d) Although we intended to perform subgroup analyses for smoking status, the dose of RT and ICIs, PD‐L1 status, oncogene driver mutation status, and different irradiated sites, current data do not support such analyses. (e) The chemotherapeutic regimens and the number of cycles administered were unclear; therefore, despite subgroup analyses, we could not eliminate the effect of chemotherapy.

## 5 CONCLUSION

Combination therapy using PD‐1/PD‐L1 inhibitors and RT for the treatment of advanced NSCLC may improve OS, PFS, and tumor response rates without an increase in ≥grade 3 AEs. However, this combination therapy increased the incidence of mild (grade 1–2) pneumonitis. The sequence of administration of PD‐1/PD‐L1 inhibitors after RT and precision radiotherapy techniques such as SBRT may offer greater benefit in patients who receive this combination therapy. Further large‐scale RCTs are warranted confirm these results.

## 6 DECLARATION

The data used to support the findings of this study are available from the corresponding author upon request. No ethic approval was required as all data originated from previously published studies.

## CONFLICTS OF INTEREST

The authors have no conflict of interest.

## AUTHORS’ CONTRIBUTIONS

Xiaohu Wang and Bing Lu contributed to choose research directions.Yichao Geng finished the manuscript. Qiuning Zhang and Zheng Li assessed the level of evidence. Ruifeng Liu and Hongtao Luo searched electronic database. Shuangwu Feng, Chengcheng Li, Lina Wang, Xueshan Zhao and Zhen Yang contributed to screened literatures. All authors read and approved the final manuscript.

## Supporting information

Supplement 1

Supplement 2

## Data Availability

I confirm that my article contains a Data Availability Statement even if no data is available (list of sample statements) unless my article type does not require one. I confirm that I have included a citation for available data in my references section, unless my article type is exempt.
