# Supplementary material for: Safety and Efficacy of PD‐1/PD‐L1 inhibitors combined with radiotherapy in patients with non‐small‐cell lung cancer: a systematic review and meta‐analysis
Source: Cancer Med. 2021 Jan 19;10(4):1222–39. doi: 10.1002/cam4.3718 (PMC7926021; doi:10.1002/cam4.3718)
Supplement: Supplementary file 1 — Supplement 1 [file CAM4-10-1222-s001.docx]

**Supplement 1: Search strategy**

1. **Pubmed search strategy**

#1 "Carcinoma, Non-Small-Cell Lung"[Mesh]

#2 (((((Non-Small-Cell Lung Carcinomas) OR Nonsmall Cell Lung Cancer) OR Non-Small-Cell Lung Carcinoma) OR Non Small Cell Lung Carcinoma) OR Non-Small Cell Lung Cancer)

#3 ("Carcinoma, Non-Small-Cell Lung"[Mesh] OR (((((Non-Small-Cell Lung Carcinomas) OR Nonsmall Cell Lung Cancer) OR Non-Small-Cell Lung Carcinoma) OR Non Small Cell Lung Carcinoma) OR Non-Small Cell Lung Cancer)

#4 "Chemoradiotherapy"[Mesh]

#5 ((("Chemoradiotherapy"[Mesh]) OR Chemoradio*) OR Radiochemo*)

#6 "Radiotherapy"[Mesh]

#7 ((((((((Radiotherap*) OR Radiation Therap*) OR Radiation Treatment*) OR irradiation) OR SABR) OR SBRT) OR SRS) OR SRT)

#8 ("Radiotherapy"[Mesh]) OR ((((((((Radiotherap*) OR Radiation Therap*) OR Radiation Treatment*) OR irradiation) OR SABR) OR SBRT) OR SRS) OR SRT)

#9 ((("Chemoradiotherapy"[Mesh]) OR Chemoradio*) OR Radiochemo*) OR ("Radiotherapy"[Mesh]) OR ((((((((Radiotherap*) OR Radiation Therap*) OR Radiation Treatment*) OR irradiation) OR SABR) OR SBRT) OR SRS) OR SRT)

#10 (((((((((((((((((((((((((((((((((((Programmed death-1) OR PD-1) OR PD1) OR Programmed death ligand-1) OR PD-L1) OR PDL1) OR checkpoint inhibitor) OR checkpoint blockade) OR nivolumab) OR opdivo) OR ono-4538) OR MDX-1106) OR BMS-936558) OR nivo) OR pembrolizumab) OR lambrolizumab) OR keytruda) OR SCH 900475) OR MK-3475) OR atezolizumab) OR MSB0010718C) OR tecentriq) OR RO5541267) OR RG7446) OR MPDL3280A) OR durvalumab) OR imfinzi) OR MEDI-4736) OR MEDI4736) OR avelumab) OR barvencik) OR MSB0010718C) OR cemiplimab) OR libtayo) OR REGN2810)))

#11 ("Carcinoma, Non-Small-Cell Lung"[Mesh] OR (((((Non-Small-Cell Lung Carcinomas) OR Nonsmall Cell Lung Cancer) OR Non-Small-Cell Lung Carcinoma) OR Non Small Cell Lung Carcinoma) OR Non-Small Cell Lung Cancer) AND ((("Chemoradiotherapy"[Mesh]) OR Chemoradio*) OR Radiochemo*) OR ("Radiotherapy"[Mesh]) OR ((((((((Radiotherap*) OR Radiation Therap*) OR Radiation Treatment*) OR irradiation) OR SABR) OR SBRT) OR SRS) OR SRT) AND (((((((((((((((((((((((((((((((((((Programmed death-1) OR PD-1) OR PD1) OR Programmed death ligand-1) OR PD-L1) OR PDL1) OR checkpoint inhibitor) OR checkpoint blockade) OR nivolumab) OR opdivo) OR ono-4538) OR MDX-1106) OR BMS-936558) OR nivo) OR pembrolizumab) OR lambrolizumab) OR keytruda) OR SCH 900475) OR MK-3475) OR atezolizumab) OR MSB0010718C) OR tecentriq) OR RO5541267) OR RG7446) OR MPDL3280A) OR durvalumab) OR imfinzi) OR MEDI-4736) OR MEDI4736) OR avelumab) OR barvencik) OR cemiplimab) OR libtayo) OR REGN2810)))

1. **EMBASE search strategy**

#1 'non small cell lung cancer'/exp

#2 'non small cell lung carcinomas':ti,ab,kw OR 'non small cell lung cancer':ti,ab,kw OR 'non small cell lung carcinoma':ti,ab,kw OR 'nsclc':ti,ab,kw

#3 #1 OR #2

#4 'chemoradiotherapy'/exp

#5 'radiotherapy'/exp

#6 chemoradio* OR radiochemo* OR radiotherap* OR radiation therap* OR radiation treatment* OR 'irradiation':ti,ab,kw OR 'sabr':ti,ab,kw OR 'sbrt':ti,ab,kw, OR 'srs':ti,ab,kw, OR 'srt':ti,ab,kw

#7 #4 OR #5 OR #6

#8 'programmed death 1 ligand 1'/exp

#9 'programmed death 1 receptor'/exp

#10 'pd1'/exp

#11 'pdl1'/exp

#12 'programmed death 1':ti,ab,kw OR 'programmed death ligand 1':ti,ab,kw OR 'checkpoint inhibitor':ti,ab,kw OR 'checkpoint blockade':ti,ab,kw OR 'nivolumab':ti,ab,kw OR 'opdivo':ti,ab,kw OR 'ono4538':ti,ab,kw OR 'mdx1106':ti,ab,kw OR 'bms936558':ti,ab,kw OR 'nivo':ti,ab,kw OR 'pembrolizumab':ti,ab,kw OR 'lambrolizumab':ti,ab,kw OR 'keytruda':ti,ab,kw OR 'sch900475':ti,ab,kw OR 'mk3475':ti,ab,kw OR 'atezolizumab':ti,ab,kw OR 'msb0010718c':ti,ab,kw OR 'tecentriq':ti,ab,kw OR 'ro5541267':ti,ab,kw OR rg7446':ti,ab,kw OR 'mpdl3280a':ti,ab,kw OR 'durvalumab':ti,ab,kw OR 'imfinzi':ti,ab,kw OR 'medi4736':ti,ab,kw OR 'avelumab':ti,ab,kw OR 'barvencik':ti,ab,kw OR 'cemiplimab':ti,ab,kw OR 'libtayo':ti,ab,kw OR 'regn2810':ti,ab,kw

#13 #8 OR #9 OR #10 OR #11 OR #12

#14 #3 AND #7 AND #13

1. **Cochrane Library search strategy**

#1 MeSH descriptor: [Carcinoma, Non-Small-Cell Lung] explode all trees

#2 (Carcinoma, Non-Small-Cell Lung):ti,ab,kw OR (Non-Small-Cell Lung Carcinomas):ti,ab,kw OR (Non Small Cell Lung Carcinoma):ti,ab,kw (Word variations have been searched)

#3 #1 OR #2

#4 MeSH descriptor: [Chemoradiotherapy] explode all trees

#5 MeSH descriptor: [Radiotherapy] explode all trees

#6 Chemoradio* OR Radiochemo* OR Radiotherap* OR Radiation Therap*OR Radiation Treatment*OR (irradiation):ti,ab,kw OR (SABR):ti,ab,kw OR (SBRT):ti,ab,kw OR (SRS):ti,ab,kw OR (SRT):ti,ab,kw (Word variations have been searched)

#7 #4 OR #5 OR #6

#8 MeSH descriptor: [Programmed Cell Death 1 Receptor] explode all trees

#9 (Programmed death-1):ti,ab,kw OR (PD-1):ti,ab,kw OR (PD1):ti,ab,kw OR (Programmed death ligand-1):ti,ab,kw OR (PD-L1):ti,ab,kw OR (PDL1):ti,ab,kw OR (checkpoint inhibitor):ti,ab,kw OR (checkpoint blockade):ti,ab,kw OR (nivolumab):ti,ab,kw OR (opdivo):ti,ab,kw OR (ono-4538):ti,ab,kw OR (MDX-1106):ti,ab,kw OR (BMS-936558):ti,ab,kw OR (nivo):ti,ab,kw OR (pembrolizumab):ti,ab,kw OR (lambrolizumab):ti,ab,kw OR (keytruda):ti,ab,kw OR (SCH 900475):ti,ab,kw OR (MK-3475):ti,ab,kw OR (atezolizumab):ti,ab,kw OR (MSB0010718C):ti,ab,kw OR (tecentriq):ti,ab,kw OR (RO5541267):ti,ab,kw OR (RG7446):ti,ab,kw OR (MPDL3280A):ti,ab,kw OR (durvalumab):ti,ab,kw OR (imfinzi):ti,ab,kw OR (MEDI-4736):ti,ab,kw OR (MEDI4736):ti,ab,kw OR (avelumab):ti,ab,kw OR (barvencik):ti,ab,kw OR (cemiplimab):ti,ab,kw OR (libtayo):ti,ab,kw OR (REGN2810):ti,ab,kw(Word variations have been searched)

#10 #8 OR #9

#11 #3 AND #7 AND #10
