# Supplementary material for: Safety and Efficacy of PD‐1/PD‐L1 inhibitors combined with radiotherapy in patients with non‐small‐cell lung cancer: a systematic review and meta‐analysis
Source: Cancer Med. 2021 Jan 19;10(4):1222–39. doi: 10.1002/cam4.3718 (PMC7926021; doi:10.1002/cam4.3718)
Supplement: Supplementary file 2 — Supplement 2 [file CAM4-10-1222-s002.docx]

**Supplement 2**

**Table S1** Quality assessment of the included RCTs

**Table S2** Quality assessment of the included cohort or case-control studies

**Table S3** The Case Series Report Quality Evaluation Form for the included single arm studies

**Table S4** The meta-analysis and subgroup analyses of the 2-years OS

**Table S5** The meta-analysis and subgroup analyses of the 1-year OS

**Table S6** The meta-analysis and subgroup analyses of the 0.5-year OS.

**Table S7** The meta-analysis and subgroup analyses of the 1-2year PFS.

**Table S8** The meta-analysis and subgroup analyses of the 0.5-year PFS.

**Table S9** The meta-analysis and subgroup analyses of ORR and DCR.

**Table S10** The meta-analysis and subgroup analyses of the adverse events.

**Table S11** The meta-analysis and subgroup analyses of the pneumonitis.

**Table S12** Meta-analysis of the cerebral radiation necrosis for the RT+ICI group compared with the RT group

**Table S1** Quality assessment of the included RCTs

| Author | Year | Random sequence generation | Allocation concealment | Blinding of participants and personnel | Blinding of outcome assessment | Incomplete outcome data | Selective reporting | Other bias |
| --- | --- | --- | --- | --- | --- | --- | --- | --- |
| Theelen | 2019 | low risk | unclear risk | low risk | low risk | low risk | low risk | unclear risk |
| Gray | 2020 | low risk | low risk | low risk | low risk | low risk | low risk | unclear risk |

**Table S2** Quality assessment of the included cohort or case-control studies

| Study | Selection | | | |  | Comparability |  | Outcome | | | Quality score |
| --- | --- | --- | --- | --- | --- | --- | --- | --- | --- | --- | --- |
|  | 1 | 2 | 3 | 4 |  | 1 |  | 1 | 2 | 3 |  |
| Shaverdian 2017 | * | * | * | * |  | ** |  | * | * | * | 9 |
| Tamiya 2017 | * | * | * | * |  |  |  | * | * | * | 7 |
| Fiorica 2018 | * | * | * | * |  | ** |  | * | * | * | 9 |
| Hubbeling 2018 | * | * | * | * |  | ** |  | * | * | * | 9 |
| Shepard 2019 | * | * | * | * |  | ** |  | * | * | * | 9 |
| Yamaguchi 2019 | * | * | * | * |  | ** |  | * | * | * | 9 |
| Ratnayake 2020 | * | * | * | * |  | ** |  | * | * | * | 9 |
| Fukui 2020 | * | * | * | * |  |  |  | * | * | * | 7 |
| Singh 2020 | * | * | * | * |  | ** |  | * | * | * | 9 |

Points were awarded for patient selection (maximum 4 points), the comparability of cohorts (maximum 2 points) and outcome assessment (maximum 3 points) and summed for an overall quality rating with a maximum of 9 points .

**Table S3** The Case Series Report Quality Evaluation Form for the included single arm studies

|  | 1 | 2 | 3 | 4 | 5 | 6 | 7 | 8 |
| --- | --- | --- | --- | --- | --- | --- | --- | --- |
| Ahmed 2017 | NO | No clear | YES | YES | YES | YES | YES | Unapplicable |
| Lesueur 2018 | YES | YES | YES | YES | YES | YES | YES | Unapplicable |
| Schapira 2018 | NO | YES | YES | YES | YES | YES | YES | Unapplicable |
| Miyamoto 2019 | YES | YES | YES | YES | YES | NO | YES | Unapplicable |
| Qin 2019 | YES | YES | YES | YES | YES | NO | YES | Unapplicable |
| Amino 2020 | No clear | YES | YES | YES | YES | No clear | YES | Unapplicable |
| Chu 2020 | No clear | YES | YES | YES | YES | NO | YES | Unapplicable |
| Jabbour 2020 | YES | YES | YES | YES | YES | YES | YES | Unapplicable |
| Miura 2020 | No clear | YES | YES | YES | YES | No clear | YES | Unapplicable |

With yes, no, and unclear. Given that this evaluation tool with twenty-three items is too cumbersome, this paper is streamlined to eight. The evaluation indicators follow: 1. Inclusion criteria and exclusion criteria; 2. Clinical heterogeneity of patients, including the severity, classification, duration and onset time of the disease; 3. Whether the main intervention measures are clearly described (dose, administration and course of treatment, etc.); 4. Whether the measurement method of relevant outcome measures is reasonable; 5. Whether the outcome measures are measured before and after the intervention; 6. Whether the loss to follow-up and follow-up time are reported; 7. Whether the occurrence of adverse events related to clinical treatment is reported; 8. Whether the outcome measurer is blinded Literature quality.

**Table S4** The meta-analysis and subgroup analyses of the 2-year OS

| Outcomes | Type of study | No. of trials | Effects model | OR(95% CI) | P-Value | Tests for heterogeneity | | Cross P-value |
| --- | --- | --- | --- | --- | --- | --- | --- | --- |
|  |  |  |  |  |  | *I^2^*, % | P-Value |  |
| **2-yOS** | Overall | 4 | M-H, Random | 1.90(1.06, 3.40) | 0.031* | 56.3% | 0.076 | - |
| Study types | NRCT | 3 | M-H, Random | 2.50(0.81, 7.66) | 0.110 | 70.6% | 0.033 | 0.673 |
|  | RCT | 1 | M-H, Random | 1.60(1.16, 2.20) | 0.004** | - | - |  |
| RT sites | RT of metastases | 2 | M-H, Random | 1.41(0.79, 2.53) | 0.251 | 0.0% | 0.541 | 0.540 |
|  | Thoracic RT | 2 | M-H, Random | 3.72(0.54, 25.93) | 0.184 | 83.7% | 0.013 |  |
| Comparison groups | RT+ICIs vs ICIs | 2 | M-H, Random | 1.41(0.79, 2.53) | 0.251 | 0.0% | 0.541 | 0.540 |
|  | RT+ICIs vs RT | 2 | M-H, Random | 3.72(0.54, 25.93) | 0.184 | 83.7% | 0.013 |  |
| Techniques | SRS or SBRT included | 2 | M-H, Random | 1.41(0.79, 2.53) | 0.251 | 0.0% | 0.541 | 0.540 |
|  | Conventional RT | 2 | M-H, Random | 3.72(0.54, 25.93) | 0.184 | 83.7% | 0.013 |  |
| Sequence | Sequential(RT first) | 4 | M-H, Random | 1.90(1.06, 3.40) | 0.031* | 56.3% | 0.076 | - |
|  | Concurrent or  Sequential(ICIs first) | 0 | M-H, Random | - | - | - | - |  |
| ICIs types | PD-1 | 2 | M-H, Random | 1.41(0.79, 2.53) | 0.251 | 0.0% | 0.541 | 0.040 |
|  | PD-L1 | 1 | M-H, Random | 1.60(1.16, 2.20) | 0.004** | - | - |  |
|  | PD-1 or PD-L1 | 1 | M-H, Random | 11.69(2.48, 55.22) | 0.002** | - | - |  |
| Stage | III | 2 | M-H, Random | 3.72(0.54, 25.93) | 0.184 | 83.7% | 0.013 | 0.688 |
|  | III-IV | 1 | M-H, Random | 1.24(0.61, 2.53) | 0.549 | - | - |  |
|  | IV | 1 | M-H, Random | 1.84(0.65, 5.16) | 0.249 | - | - |  |
| Brain metastases | Yes | 2 | M-H, Random | 1.41(0.79, 2.53) | 0.251 | 0.0% | 0.541 | 0.540 |
|  | No | 2 | M-H, Random | 3.72(0.54, 25.93) | 0.184 | 83.7% | 0.013 |  |

Abbreviation: RT radiotherapy, ICIs Immune checkpoint inhibitors, OS overall survival, RCT randomized controlled trial, NRCT non-randomized controlled trial, SRS stereotatic radiosurgery,

SBRT stereotactic body radiation therapy

*0.01<P<0.05, **0.001<P<0.01

**Table S5** The meta-analysis and subgroup analyses of the 1-year OS

| Outcomes | Type of study | No. of trials | Effects model | OR(95% CI) | P-Value | Tests for heterogeneity | | Cross P-value |
| --- | --- | --- | --- | --- | --- | --- | --- | --- |
|  |  |  |  |  |  | *I^2^*, % | P-Value |  |
| **1-yOS** | Overall | 8 | M-H, Random | 1.77(1.35, 2.33) | 0.000*** | 0.0% | 0.534 | - |
| Study types | NRCT | 6 | M-H, Random | 1.82(1.14, 2.93) | 0.013* | 11.6% | 0.341 | 0.936 |
|  | RCT | 2 | M-H, Random | 1.77(1.35, 2.33) | 0.002** | 0.0% | 0.524 |  |
| RT sites | RT of metastases | 6 | M-H, Random | 1.83(1.23, 2.72) | 0.003** | 0.0% | 0.430 | 0.946 |
|  | Thoracic RT | 2 | M-H, Random | 1.88(0.85, 4.16) | 0.122 | 11.3% | 0.288 |  |
| Comparison groups | RT+ICIs vs ICIs | 5 | M-H, Random | 2.07(1.36, 3.15) | 0.001** | 0.0% | 0.743 | 0.340 |
|  | RT+ICIs vs RT | 3 | M-H, Random | 1.46(0.67, 3.17) | 0.343 | 37.2% | 0.203 |  |
| Techniques | SRS or SBRT included | 6 | M-H, Random | 1.83(1.23, 2.72) | 0.003* | 0.0% | 0.430 | 0.825 |
|  | Conventional RT | 2 | M-H, Random | 1.88(0.85, 4.16) | 0.122 | 11.3% | 0.288 |  |
| Sequence | Sequential(RT first) | 6 | M-H, Random | 1.84(1.38, 2.45) | 0.000*** | 0.0% | 0.661 | 0.445 |
|  | Concurrent or Sequential(ICIs first) | 2 | M-H, Random | 1.31(0.37, 4.60) | 0.672 | 55.0% | 0.136 |  |
| ICIs types | PD-1 | 5 | M-H, Random | 2.07(1.36, 3.15) | 0.001** | 0.0% | 0.743 | 0.423 |
|  | PD-L1 | 1 | M-H, Random | 1.68(1.14, 2.45) | 0.007** | - | - |  |
|  | PD-1 or PD-L1 | 2 | M-H, Random | 1.70(0.15, 19.44) | 0.671 | 62.3% | 0.104 |  |
| Stage | III | 2 | M-H, Random | 1.88(0.85, 4.16) | 0.122 | 11.3% | 0.288 | 0.969 |
|  | III-IV | 2 | M-H, Random | 2.17(0.80, 5.86) | 0.127 | 42.1% | 0.189 |  |
|  | IV | 4 | M-H, Random | 1.79(1.07, 3.00) | 0.026* | 4.6% | 0.370 |  |
| Brain metastases | Yes | 4 | M-H, Random | 1.58(1.00, 2.50) | 0.051 | 0.0% | 0.432 | 0.537 |
|  | No | 4 | M-H, Random | 1.89(1.35, 2.65) | 0.000*** | 0.0% | 0.402 |  |

Abbreviation: RT radiotherapy, ICIs Immune checkpoint inhibitors, OS overall survival, RCT randomized controlled trial, NRCT non-randomized controlled trial, SRS stereotatic radiosurgery,

SBRT stereotactic body radiation therapy

*0.01<P<0.05, **0.001<P<0.01, ***P<0.001

**Table S6** The meta-analysis and subgroup analyses of the 0.5-year OS.

| Outcomes | Type of study | No. of trials | Effects model | OR(95% CI) | P-Value | Tests for heterogeneity | | Cross P-value |
| --- | --- | --- | --- | --- | --- | --- | --- | --- |
|  |  |  |  |  |  | *I^2^*, % | P-Value |  |
| **0.5-yOS** | Overall | 6 | M-H, Random | 1.49(0.72, 3.09) | 0.281 | 66.7% | 0.010 | - |
| Study types | NRCT | 5 | M-H, Random | 1.43(0.58, 3.53) | 0.436 | 73.3% | 0.005 | 0.815 |
|  | RCT | 1 | M-H, Random | 1.73(0.66, 4.55) | 0.264 | - | - |  |
| RT sites | RT of metastases | 6 | M-H, Random | 1.49(0.72, 3.09) | 0.281 | 66.7% | 0.010 | - |
|  | Thoracic RT | 0 | - | - | - | - | - |  |
| Comparison groups | RT+ICIs vs ICIs | 5 | M-H, Random | 1.96(1.22, 3.15) | 0.006** | 17.3% | 0.304 | 0.001 |
|  | RT+ICIs vs RT | 1 | M-H, Random | 0.19(0.05, 0.74) | 0.017* | - | - |  |
| Techniques | SRS or SBRT included | 6 | M-H, Random | 1.49(0.72, 3.09) | 0.281 | 66.7% | 0.010 | - |
|  | Conventional RT | 0 | - | - | - | - | - |  |
| Sequence | Sequential(RT first) | 4 | M-H, Random | 1.94(1.06, 3.57) | 0.032* | 36.7% | 0.192 | 0.138 |
|  | Concurrent or Sequential(ICIs first) | 2 | M-H, Random | 0.68(0.06, 7.50) | 0.757 | 87.6% | 0.005 |  |
| ICIs types | PD-1 | 5 | M-H, Random | 1.96(1.22, 3.15) | 0.006** | 17.3% | 0.304 | 0.001 |
|  | PD-L1 | 0 | - | - | - | - | - |  |
|  | PD-1 or PD-L1 | 1 | M-H, Random | 0.19(0.05, 0.74) | 0.017* | - | - |  |
| Stage | III | 0 | - | - | - | - | - | 0.584 |
|  | III-IV | 2 | M-H, Random | 1.58(0.52, 4.83) | 0.424 | 50.3% | 0.156 |  |
|  | IV | 4 | M-H, Random | 1.38(0.48, 3.98) | 0.551 | 76.4% | 0.005 |  |
| Brain metastases | Yes | 4 | M-H, Random | 1.20(0.43, 3.40) | 0.729 | 78.1% | 0.003 | 0.381 |
|  | No | 4 | M-H, Random | 2.13(0.95, 4.75) | 0.065 | 0.0% | 0.455 |  |

Abbreviation: RT radiotherapy, ICIs Immune checkpoint inhibitors,OS overall survival, RCT randomized controlled trial, NRCT non-randomized controlled trial, SRS stereotatic radiosurgery,

SBRT stereotactic body radiation therapy

*0.01<P<0.05, **0.001<P<0.01

**Table S7** The meta-analysis and subgroup analyses of the 1-2year PFS.

| Outcomes | Type of study | No. of trials | Effects model | OR(95% CI) | P-Value | Tests for heterogeneity | | Cross P-value |
| --- | --- | --- | --- | --- | --- | --- | --- | --- |
|  |  |  |  |  |  | *I^2^*, % | P-Value |  |
| **2-yPFS** | Overall | 2 | M-H, Random | 2.47(1.13, 5.37) | 0.023* | 0.0% | 0.921 | - |
| **1-yPFS** | Overall | 6 | M-H, Random | 2.09(1.29, 3.38) | 0.003** | 45.9% | 0.100 | - |
| Study types | NRCT | 4 | M-H, Random | 2.14(0.84, 5.49) | 0.112 | 65.1% | 0.035 | 0.960 |
|  | RCT | 2 | M-H, Random | 2.22(1.63, 3.01) | 0.000*** | 0.0% | 0.420 |  |
| RT sites | RT of metastases | 5 | M-H, Random | 1.98(0.96, 4.10) | 0.065 | 55.6% | 0.061 | 0.630 |
|  | Thoracic RT | 1 | M-H, Random | 2.31(1.67, 3.18) | 0.000*** | - | - |  |
| Comparison groups | RT+ICIs vs ICIs | 4 | M-H, Random | 2.62(1.57, 4.38) | 0.000*** | 0.0% | 0.486 | 0.443 |
|  | RT+ICIs vs RT | 2 | M-H, Random | 1.18(0.26, 5.36) | 0.832 | 83.9% | 0.013 |  |
| Techniques | SRS or SBRT included | 5 | M-H, Random | 1.98(0.96, 4.10) | 0.065 | 55.6% | 0.061 | 0.630 |
|  | Conventional RT | 1 | M-H, Random | 2.31(1.67, 3.18) | 0.000*** | - | - |  |
| Sequence | Sequential(RT first) | 4 | M-H, Random | 2.62(1.57, 4.38) | 0.000*** | 0.0% | 0.486 | 0.443 |
|  | Concurrent or Sequential(ICIs first) | 2 | M-H, Random | 1.18(0.26, 5.36) | 0.832 | 83.9% | 0.013 |  |
| ICIs types | PD-1 | 4 | M-H, Random | 2.62(1.57, 4.38) | 0.000*** | 0.0% | 0.486 | 0.033 |
|  | PD-L1 | 1 | M-H, Random | 2.31(1.67, 3.18) | 0.000*** | - | - |  |
|  | PD-1 or PD-L1 | 1 | M-H, Random | 0.49(0.15, 1.58) | 0.231 | - | - |  |
| Stage | III | 1 | M-H, Random | 2.31(1.67, 3.18) | 0.000*** | - | - | 0.145 |
|  | III-IV | 2 | M-H, Random | 3.47(1.66, 7.25) | 0.001** | 0.0% | 0.415 |  |
|  | IV | 3 | M-H, Random | 1.31(0.51, 3.37) | 0.579 | 57.5% | 0.095 |  |
| Brain metastases | Yes | 3 | M-H, Random | 1.67(0.58, 4.83) | 0.347 | 69.7% | 0.037 | 0.541 |
|  | No | 3 | M-H, Random | 2.31(1.55, 3.45) | 0.000*** | 11.7% | 0.322 |  |

Abbreviation: RT radiotherapy, ICIs Immune checkpoint inhibitors, PFS progression-free survival, RCT randomized controlled trial,NRCT non-randomized controlled trial, SRS stereotatic radiosurgery,

SBRT stereotactic body radiation therapy

*0.01<P<0.05, **0.001<P<0.01, ***P<0.001

**Table S8** The meta-analysis and subgroup analyses of the 0.5-year PFS.

| Outcomes | Type of study | No. of trials | Effects model | OR(95% CI) | P-Value | Tests for heterogeneity | | Cross P-value |
| --- | --- | --- | --- | --- | --- | --- | --- | --- |
|  |  |  |  |  |  | *I^2^*, % | P-Value |  |
| **0.5-yPFS** | Overall | 7 | M-H, Random | 1.83(1.13, 2.98) | 0.014* | 43.1% | 0.104 | - |
| Study types | NRCT | 6 | M-H, Random | 1.78(1.00, 3.18) | 0.052 | 52.3% | 0.063 | 0.800 |
|  | RCT | 1 | M-H, Random | 2.08(0.83, 5.22) | 0.121 | - | - |  |
| RT sites | RT of metastases | 6 | M-H, Random | 2.02(1.16, 3.53) | 0.013* | 42.2% | 0.124 | 0.168 |
|  | Thoracic RT | 1 | M-H, Random | 1.22(0.61, 2.44) | 0.572 | - | - |  |
| Comparison groups | RT+ICIs vs ICIs | 6 | M-H, Random | 2.12(1.47, 3.06) | 0.000*** | 0.0% | 0.566 | 0.010 |
|  | RT+ICIs vs RT | 1 | M-H, Random | 0.37(0.10, 1.33) | 0.127 | - | - |  |
| Techniques | SRS or SBRT included | 7 | M-H, Random | 1.83(1.13, 2.98) | 0.014* | 43.1% | 0.104 | - |
|  | Conventional RT | 0 | - | - | - | - | - |  |
| Sequence | Sequential(RT first) | 5 | M-H, Random | 2.11(1.44, 3.08) | 0.000*** | 0.0% | 0.425 | 0.091 |
|  | Concurrent or Sequential(ICIs first) | 2 | M-H, Random | 0.92(0.15, 5.65) | 0.928 | 74.1% | 0.050 |  |
| ICIs types | PD-1 | 6 | M-H, Random | 2.12(1.47, 3.06) | 0.000*** | 0.0% | 0.566 | 0.010 |
|  | PD-L1 | 0 | - | - | - | - | - |  |
|  | PD-1 or PD-L1 | 1 | M-H, Random | 0.37(0.10, 1.33) | 0.127 | - | - |  |
| Stage | III | 0 | - | - | - | - | - | 0.185 |
|  | III-IV | 2 | M-H, Random | 2.69(1.40, 5.16) | 0.003** | 0.0% | 0.956 |  |
|  | IV | 5 | M-H, Random | 1.56(0.82, 2.97) | 0.179 | 54.5% | 0.067 |  |
| Brain metastases | Yes | 4 | M-H, Random | 1.81(0.77, 4.25) | 0.174 | 64.7% | 0.037 | 0.462 |
|  | No | 3 | M-H, Random | 1.61(0.97, 2.70) | 0.068 | 0.0% | 0.471 |  |

Abbreviation: RT radiotherapy, ICIs Immune checkpoint inhibitors, PFS progression-free survival, RCT randomized controlled trial, NRCT non-randomized controlled trial, SRS stereotatic radiosurgery,

SBRT stereotactic body radiation therapy

*0.01<P<0.05, **0.001<P<0.01, ***P<0.001

**Table S9** The meta-analysis and subgroup analyses of ORR and DCR.

| Outcomes | Type of study | No. of trials | Effects model | OR(95% CI) | P-Value | Tests for heterogeneity | | Cross P-value |
| --- | --- | --- | --- | --- | --- | --- | --- | --- |
|  |  |  |  |  |  | I2, % | P-Value |  |
| **ORR** | Overall | 5 | M-H, Random | 2.76(1.06, 7.19) | 0.038* | 78.1% | 0.001 | - |
| Study types | NRCT | 4 | M-H, Random | 2.77(0.79, 9.76) | 0.112 | 83.5% | 0.000 | 0.919 |
|  | RCT | 1 | M-H, Random | 3.08(1.15, 8.29) | 0.026* | - | - |  |
| Comparison groups | RT+ICIs vs ICIs | 3 | M-H, Random | 1.60(0.55, 4.59) | 0.387 | 73.6% | 0.023 | 0.00 |
|  | RT+ICIs vs RT | 2 | M-H, Random | 7.04(2.27, 21.87) | 0.001** | 20.0% | 0.264 |  |
| **DCR** | Overall | 5 | M-H, Random | 1.80(1.21, 2.68) | 0.004** | 0.0% | 0.560 | - |
| Study types | NRCT | 4 | M-H, Random | 1.64(1.06, 2.53) | 0.026* | 0.0% | 0.595 | 0.295 |
|  | RCT | 1 | M-H, Random | 2.87(1.10, 7.49) | 0.031* | - | - |  |
| Comparison groups | RT+ICIs vs ICIs | 3 | M-H, Random | 1.93(1.25, 2.97) | 0.003** | 0.0% | 0.400 | 0.454 |
|  | RT+ICIs vs RT | 2 | M-H, Random | 1.27(0.47, 3.44) | 0.633 | 0.0% | 0.437 |  |

Abbreviation: RT radiotherapy, ICIs Immune checkpoint inhibitors, ORR objective response rate, DCR disease control rate, RCT randomized controlled trial, NRCT non-randomized controlled trial

*0.01<P<0.05, **0.001<P<0.01

**Table S10** The meta-analysis and subgroup analyses of the adverse events.

| Outcomes | Type of study | No. of trials | Effects model | OR(95% CI) | P-Value | Tests for heterogeneity | | Cross P-value |
| --- | --- | --- | --- | --- | --- | --- | --- | --- |
|  |  |  |  |  |  | *I^2^*, % | P-Value |  |
| **AE<G3** | Overall | 3 | M-H, Random | 0.97(0.71, 1.34) | 0.858 | 0.0% | 0.544 | - |
| Study types | NRCT | 2 | M-H, Random | 1.80(0.56, 5.74) | 0.322 | 0.0% | 0.825 | 0.280 |
|  | RCT | 1 | M-H, Random | 0.92(0.66, 1.29) | 0.639 | - | - |  |
| RT sites | RT of metastases | 2 | M-H, Random | 1.80(0.56, 5.74) | 0.322 | 0.0% | 0.825 | 0.280 |
|  | Thoracic RT | 1 | M-H, Random | 0.92(0.66, 1.29) | 0.639 | - | - |  |
| Comparison groups | RT+ICIs vs ICIs | 2 | M-H, Random | 1.80(0.56, 5.74) | 0.322 | 0.0% | 0.825 | 0.280 |
|  | RT+ICIs vs RT | 1 | M-H, Random | 0.92(0.66, 1.29) | 0.639 | - | - |  |
| Techniques | SRS or SBRT included | 2 | M-H, Random | 1.80(0.56, 5.74) | 0.322 | 0.0% | 0.825 | 0.280 |
|  | Conventional RT | 1 | M-H, Random | 0.92(0.66, 1.29) | 0.639 | - | - |  |
| Sequence | Sequential(RT first) | 2 | M-H, Random | 0.95(0.69, 1.32) | 0.767 | 0.0% | 0.358 | 0.543 |
|  | Concurrent or Sequential(ICIs first) | 1 | M-H, Random | 1.59(0.32, 7.92) | 0.575 | - | - |  |
| ICIs types | PD-1 | 2 | M-H, Random | 1.80(0.56, 5.74) | 0.322 | 0.0% | 0.825 | 0.280 |
|  | PD-L1 | 1 | M-H, Random | 0.92(0.66, 1.29) | 0.639 | - | - |  |
|  | PD-1 or PD-L1 | 0 | - | - | - | - | - |  |
| **AE>=G3** | Overall | 3 | M-H, Random | 1.24(0.88, 1.74) | 0.222 | 0.0% | 0.809 | - |
| Study types | NRCT | 2 | M-H, Random | 0.84(0.20, 3.64) | 0.819 | 0.0% | 0.704 | 0.598 |
|  | RCT | 1 | M-H, Random | 1.26(0.89, 1.80) | 0.190 | - | - |  |
| RT sites | RT of metastases | 2 | M-H, Random | 0.84(0.20, 3.64) | 0.819 | 0.0% | 0.704 | 0.598 |
|  | Thoracic RT | 1 | M-H, Random | 1.26(0.89, 1.80) | 0.190 | - | - |  |
| Comparison groups | RT+ICIs vs ICIs | 2 | M-H, Random | 0.84(0.20, 3.64) | 0.819 | 0.0% | 0.704 | 0.598 |
|  | RT+ICIs vs RT | 1 | M-H, Random | 1.26(0.89, 1.80) | 0.190 | - | - |  |
| Techniques | SRS or SBRT included | 2 | M-H, Random | 0.84(0.20, 3.64) | 0.819 | 0.0% | 0.704 | 0.598 |
|  | Conventional RT | 1 | M-H, Random | 1.26(0.89, 1.80) | 0.190 | - | - |  |
| Sequence | Sequential(RT first) | 2 | M-H, Random | 1.36(0.08, 23.62) | 0.834 | - | - | 0.949 |
|  | Concurrent or Sequential(ICIs first) | 1 | M-H, Random | 1.24(0.88, 1.74) | 0.229 | 0.0% | 0.518 |  |
| ICIs types | PD-1 | 2 | M-H, Random | 0.84(0.20, 3.64) | 0.819 | 0.0% | 0.704 | 0.598 |
|  | PD-L1 | 1 | M-H, Random | 1.26(0.89, 1.80) | 0.190 | - | - |  |
|  | PD-1 or PD-L1 | 0 | - | - | - | - | - |  |

Abbreviation: RT radiotherapy, ICIs Immune checkpoint inhibitors, AE adverse events, G3 Grade 3, SRS stereotatic radiosurgery, SBRT stereotactic body radiation therapy

**Table S11** The meta-analysis and subgroup analyses of the pneumonitis.

| Outcomes | Type of study | No. of trials | Effects model | OR(95% CI) | P-Value | Tests for heterogeneity | | Cross P-value |
| --- | --- | --- | --- | --- | --- | --- | --- | --- |
|  |  |  |  |  |  | *I^2^*, % | P-Value |  |
| **Pneumonitis<G3** | Overall | 5 | M-H, Random | 1.54(1.08, 2.21) | 0.018* | 0.0% | 0.544 | - |
| Study types | NRCT | 3 | M-H, Random | 3.33(0.36, 31.05) | 0.291 | 0.0% | 0.825 | 0.493 |
|  | RCT | 2 | M-H, Random | 2.27(0.45, 11.41) | 0.322 | - | - |  |
| RT sites | RT of metastases | 4 | M-H, Random | 5.09(0.86, 30.24) | 0.074 | 0.0% | 0.566 | 0.180 |
|  | Thoracic RT | 1 | M-H, Random | 1.47(1.02, 2.12) | 0.040* | - | - |  |
| Comparison groups | RT+ICIs vs ICIs | 4 | M-H, Random | 5.09(0.86, 30.24) | 0.074 | 0.0% | 0.566 | 0.180 |
|  | RT+ICIs vs RT | 1 | M-H, Random | 1.47(1.02, 2.12) | 0.040* | - | - |  |
| Sequence | Sequential(RT first) | 4 | M-H, Random | 2.56(0.69, 9.47) | 0.158 | 31.8% | 0.231 | 0.912 |
|  | Concurrent or Sequential(ICIs first) | 1 | M-H, Random | 1.30(0.06, 28.39) | 0.868 | - | - |  |
| ICIs types | PD-1 | 4 | M-H, Random | 5.09(0.86, 30.24) | 0.074 | 0.0% | 0.566 | 0.180 |
|  | PD-L1 | 1 | M-H, Random | 1.47(1.02, 2.12) | 0.040* | - | - |  |
|  | PD-1 or PD-L1 | 0 | - | - | - | - | - |  |
| **Pneumonitis>=G3** | Overall | 5 | M-H, Random | 0.76(0.16, 3.59) | 0.724 | 52.0% | 0.100 | - |
| Study types | NRCT | 3 | M-H, Random | 0.55(0.02, 14.60) | 0.718 | 67.7% | 0.079 | 0.254 |
|  | RCT | 2 | M-H, Random | 1.00(0.14, 7.19) | 0.997 | 46.8% | 0.170 |  |
| RT sites | RT of metastases | 4 | M-H, Random | 0.39(0.05, 3.06) | 0.371 | 39.5% | 0.191 | 0.086 |
|  | Thoracic RT | 1 | M-H, Random | 1.87(0.75, 4.67) | 0.182 | - | - |  |
| Comparison groups | RT+ICIs vs ICIs | 4 | M-H, Random | 0.39(0.05, 3.06) | 0.371 | 39.5% | 0.191 | 0.086 |
|  | RT+ICIs vs RT | 1 | M-H, Random | 1.87(0.75, 4.67) | 0.182 | - | - |  |
| Sequence | Sequential(RT first) | 3 | M-H, Random | 0.85(0.06, 13.18) | 0.907 | 42.5% | 0.187 | 0.732 |
|  | Concurrent or Sequential(ICIs first) | 2 | M-H, Random | 0.58(0.04, 8.90) | 0.692 | 77.6% | 0.035 |  |
| ICIs types | PD-1 | 4 | M-H, Random | 0.39(0.05, 3.06) | 0.371 | 39.5% | 0.191 | 0.086 |
|  | PD-L1 | 1 | M-H, Random | 1.87(0.75, 4.67) | 0.182 | - | - |  |
|  | PD-1 or PD-L1 | 0 | - | - | - | - | - |  |

Abbreviation: RT radiotherapy, ICIs Immune checkpoint inhibitors,AE adverse events, G3 Grade 3, SRS stereotatic radiosurgery, SBRT stereotactic body radiation therapy

*0.01<P<0.05

**Table S12** Meta-analysis of the cerebral radiation necrosis for the RT+ICI group compared with the RT group

| Outcomes | Type of study | No. of trials | Effects model | OR(95% CI) | P-Value | Tests for heterogeneity | | Cross P-value |
| --- | --- | --- | --- | --- | --- | --- | --- | --- |
|  |  |  |  |  |  | *I^2^*, % | P-Value |  |
| cerebral radiation necrosis | Overall | 3 | M-H, Random | 0.751(0.232, 2.433) | 0.634 | 0.0% | 0.799 | ─ |
